# Analysis of lncRNA sequences: *FAM3D-AS1*, *LINC01230*, *LINC01315* and *LINC01468* in endometrial cancer

**DOI:** 10.1186/s12885-022-09426-2

**Published:** 2022-03-29

**Authors:** Jan Bieńkiewicz, Hanna Romanowicz, Bożena Szymańska, Daria Domańska-Senderowska, Miłosz Wilczyński, Anna Stepowicz, Andrzej Malinowski, Beata Smolarz

**Affiliations:** 1 Department of Operative Gynecology, Endoscopy and Gynecologic Oncology, Polish Mother’s Memorial Hospital - Research Institute, 281/289, Rzgowska Street, 93-338 Lodz, Poland; 2 Department of Clinical Pathology, Polish Mother’s Memorial Hospital - Research Institute, Lodz, Poland; 3 Research Laboratory CoreLab, Medical University of Lodz, Lodz, Poland; 4 Department of Molecular Bases of Medicine, Medical University of Lodz, Lodz, Poland; 5 Department of Obstetrics, Perinatology and Gynecology, Polish Mother’s Memorial Hospital - Research Institute, Lodz, Poland; 6 Department of Operative and Endoscopic Gynecology, Medical University of Lodz, Lodz, Poland

**Keywords:** Long non-coding RNA, lncRNA, Endometrial cancer, *FAM3D-AS1*, *LINC01230*, *LINC01315*, *LINC01468*

## Abstract

**Background:**

The analysis of long non-coding RNA (lncRNA) in endometrial cancer is a novel field of science. Although numerous lncRNA sequences have been identified until today, their correlation with endometrial cancer is still undetermined. The aim of this study was to analyze the expression of four lncRNA sequences: *FAM3D-AS1*, *LINC01230*, *LINC01315* and *LINC01468* and to investigate their significance in endometrial cancer.

**Methods:**

LncRNA sequences were investigated in paraffin blocks (tumor tissue and non-malignant endometrial tissue in archival postoperative specimens) in endometrial cancer patients (Cases, *n* = 120) and in cancer-free controls (*n* = 80) using real-time PCR assay.

**Results:**

This study revealed a lower expression of *LINC01468* in endometrial cancer patients than in controls. Both *LINC01468* and *FAM3D-AS1* were positively correlated with Body Mass Index (BMI) in cancer-free controls.

**Conclusions:**

LncRNA *LINC01468* may be a protective factor in development of endometrial cancer.

**Supplementary Information:**

The online version contains supplementary material available at 10.1186/s12885-022-09426-2.

## Background

Endometrial cancer (EC) is the second most common malignancy of female genitals worldwide after cervical cancer. According to IARC (International Agency for Research on Cancer), in 2020 only more than 400,000 cases and almost 100,000 deaths were reported [1]. As this malignancy poses a serious threat to global health, it has gained global research attention: almost 14,000 original articles on this matter were published from 1900 to 2020 and this number increases continuously [2]. During the greater share of that period scholars’ understanding of EC was strictly constrained and dictated by histopathological representation of the disease: type I endometrioid tumors were associated with estrogen excess, obesity and hormone-receptor positivity, whereas type II non-endometrioid ones (predominantly serous) were less common, lacked hormone-dependency and were labelled with rather a poor prognosis. This classical perception of EC was then challenged in 2013 by a notable paper published in Nature when a completely new light shed on the disease shifted the recognition of EC from histology to genetics with even a thorough reclassification proposed [3].

With the introduction of genetic approach to cancer – as the shift towards genetics is not limited to EC only – some novel research pathways came into exploration, including the analysis of various aspects of human genome and their potential role in carcinogenesis. It is now clear that only less than 2% of DNA is represented by mRNA and bears a protein-coding potential which suggests that genes ‘operate’ in a so called ‘plethora of junk DNA’ [4]. The vast majority of human genome is transcribed into a ‘dark matter’ which was initially believed to be only a ‘transcriptional noise’. However, these inter-genic regions proved to have a considerable significance and have rightly drawn major scientific enthusiasm in recent years [5, 6]. The beforementioned ‘dark matter’ includes, among many others, long non-coding RNA (lncRNA) which is defined as transcripts greater than 200 nucleotides with no evident ability of being translated into protein. Although the role of lncRNA has not been fully elucidated yet, it is well known that it plays a vital part in numerous biological phenomena like epigenetic regulation, genetic imprinting, cell apoptosis and differentiation and even cell cycle control system [7–13]. Moreover, it regulates RNA maturation, intercellular transport and formation of ribonucleoprotein complexes [6].

It has been also demonstrated, that lncRNA may promote the growth of endometrial tissue outside the uterine cavity and thus it may contribute to development of endometriosis [14]. According to the growing body of literature, primary differences in lncRNA expression have also been found in various types of cancer [6, 15–18]. Likewise, in EC it has been concluded that some of these non-coding transcripts may be implemented as both diagnostic and prognostic biomarkers of this malignancy [4, 19, 20].

The first in-depth paper on various subtypes of lncRNA in EC was published by Zhai et al. in 2015 [21]. In this study authors have identified 53 distinct lncRNA sequences which expressions statistically significantly differed in carcinomatous endometrium and cancer-free one. Variations in expression concerned also signaling pathways, cell components and numerous other molecular features.

Recent literature data suggest that the following lncRNA sequences are associated with endometrial cancer: *ASLNC04080* (*SNHG12* – small nucleolar RNA host gene 12) [21], *H19* (human H19 gene) [22–24], *OVAAL* (ovarian adenocarcinoma amplified lncRNA) [25], *CASC2* (cancer susceptibility candidate 2) [26], *MALAT1* (metastasis-associated lung adenocarcinoma transcript 1) [27], *HOTAIR* (HOX transcript antisense RNA) [28], *SRA* (steroid receptor RNA activator) [29], *Linc-RoR* (long intergenic non-coding ribonucleic acids-regulator of programming) [30], *LIN00261* [31], *LIN00672* [32], *7-lncRNA* [33], and many others. It has also been proven, that long non-coding transcripts play some role in proliferation and migration of endometrial cancer cells [34]. Some authors even suggest employing a special algorithm that clusters specific triplets of mRNA, lncRNA and miRNA to better identify the risk of this cancer which may also reflect in deeper understanding of transcriptome regulatory mechanisms in this disease [35].

Although, as presented above, numerous lncRNA sequences are somehow correlated with EC, the exact meaning and context of this interaction remains unclear and calls for elucidation. This paper is designed to expand the still vague and incomplete knowledge of the role of selected lncRNA sequences in EC.

Following lncRNA sequences have been selected for the study: *FAM3D-AS1*, *LINC01230*, *LINC01315* and *LINC01468*. There is limited literature data on these lncRNAs, and until now none of them has been studied in endometrial cancer. Current evidence on lncRNA sequences *FAM3D-AS1* (FAM3D Antisense RNA 1) and *LINC01315* is scarce, but they have been affiliated with certain types of cancer (colon, larynx, pharynx, thyroid, breast, oral squamous and nasopharyngeal) [36–41]. LncRNA *LINC01468* (also known as *LNCAROD* - lncRNA activating regulator of DKK1), on the other hand, has been linked to head and neck cancer [42], liver cancer [43] and bladder cancer [44]. The knowledge on lncRNA *LINC01230* is rather narrow: it has been reported that is a novel modifier of endothelial function regulated by regulated by PPARγ [45], but no reports on its role in carcinogenesis are to be found.

## Methods

### Aim of the study

The aim of this study was to analyze the level of expression of four long non-coding RNA sequences (*FAM3D-AS1*, *LINC01230*, *LINC01315* and *LINC01468)* in endometrial cancer and to correlate the findings with both clinical and pathological data and with the risk of endometrial cancer.

### Patients

Biological material was retrieved from archival post-operative specimens. Endometrial cancer tissue samples (i.e. tumor tissue embedded in paraffin blocks) were obtained from 120 women (Cases) treated surgically for endometrioid EC in the Department of Operative Gynecology, Endoscopy and Gynecologic Oncology, Polish Mother’s Memorial Hospital - Research Institute, Lodz, Poland in the period between 2003 and 2012. Patients were staged according to FIGO [46] and tumor grading (G1, G2, G3) was also assessed. Similarly, endometrial tissue samples (i.e. endometrial tissue embedded in paraffin blocks) from 80 cancer-free women treated surgically for symptomatic uterine fibroids in the abovementioned Institution were selected as Controls. Histopathological analysis of all specimens was performed in Department of Clinical Pathology, Polish Mother’s Memorial Hospital - Research Institute, Lodz, Poland. Genetic assays of *FAM3D-AS1*, *LINC01230*, *LINC01315* and *LINC01468* were performed in Research Laboratory CoreLab, Medical University of Lodz, Lodz, Poland.

### Total RNA isolation

Total RNA was extracted from FFPE (Formalin-Fixed Paraffin-Embedded) tissue samples using High Pure miRNA Isolation Kit (Roche Diagnostics GmbH, Mannheim, Germany), as previously described by Wilczyński et al. [47]. In brief, FFPE microsamples were processed in 2 ml Eppendorf tubes, deparaffinized with 100% xylene, washed in 100% ethanol and dried at 55 °C for 10 min. Then, the dried tissue was resuspended in 100 μl Paraffin Tissue Lysis Buffer (included in the kit) and digested overnight with proteinase K at 55 °C. Subsequent steps of RNA purification were performed according to the 1-Column protocol for total RNA purification. Subsequently, 325 μl of Binding Buffer and 325 μl of Binding Enhancer was added and the mixture applied onto the columns, centrifuged for 30 s at 13000 x g and washed twice with 500 μl and 300 μl of Wash Buffer. An additional step of centrifugation for 1 min at 13000 x g was carried out in order to dry filter fleece completely and RNA was eluted with 50 μl of Elution Buffer. The yield and quality (260/280 optical density ratios) of the RNA products were determined using PicoDrop spectrophotometer (Picodrop Limited, Hinxton, UK). The purified total RNA was immediately used for cDNA synthesis or stored at − 80 °C until use.

### Reverse transcription

Reverse transcription was carried out using the Maxima First Strand cDNA Synthesis Kit (Thermo Fisher Scientific, Inc., Waltham, MA, USA) according to the manufacturer’s protocols. 500 ng of total RNA was used as starting material, reverse transcription was performed in conditions optimized for use with this kit (25 °C for 10 min, 50 °C for 30 min, 85 °C for 5 min). The cDNA samples were kept frozen at − 20 °C.

### Quantification of lncRNA

LncRNA quantification was done using TaqMan™ Non-coding RNA Assay *FAM3D-AS1* (lnc-KCTD6–3, Assay ID: Hs04938334_g1), TaqMan™ Gene Expression Assay *LINC01230* (linc-DMRT2; lnc-DRMT2; TCONS_00015639, Assay ID Hs05032977_s1), *LINC01315* (lnc-C22orf32–1, Assay ID: Hs01674425_m1), *LINC01468* (lnc-MBL2–4, Assay ID: Hs01380812_m1. In addition, Gapdh Assay (ID: Hs99999905_m1) was used as an endogenous control. qPCR reactions were performed in 10 μl reaction volumes included 10 ng cDNA, 5 μl TaqMan Fast Advanced PCR Master Mix and 0,5 μl appropriate assay (20x). The reactions were incubated in a 96-well plate at 95 °C for 3 min, followed by 40 cycles of 95 °C for 1 s and 60 °C for 20 s. All reactions were performed in duplicate using a 7900HT Fast Real-Time PCR System (Applied Biosystems; Thermo Fisher Scientific, Inc., Waltham, MA, USA). Relative expression level was determined using to the 2-ΔΔCq method.

### Statistical analysis

In order to process the results of lncRNA sequences’ expression, Applied Biosystems DataAssist Software V3.01 Thermo Fisher Scientific – US (https://www.thermofisher.com/pl/en/home/technical-resources/software-downloads/dataassist-software.html) was employed. The significance of differences was analyzed at the level of gene expression and mRNA, using non-parametric tests (the U Mann-Whitney test and the Kruskal-Wallis test) for a lack of distribution normality of the obtained results, as confirmed by the Shapiro-Wilk test. The R-Spearman test was used to assess the correlations between variables. A statistical significance was confirmed at *p* < 0,05. Statistica 13.3. software was used for statistical analysis of obtained data.

## Results

**Fig. 1:**
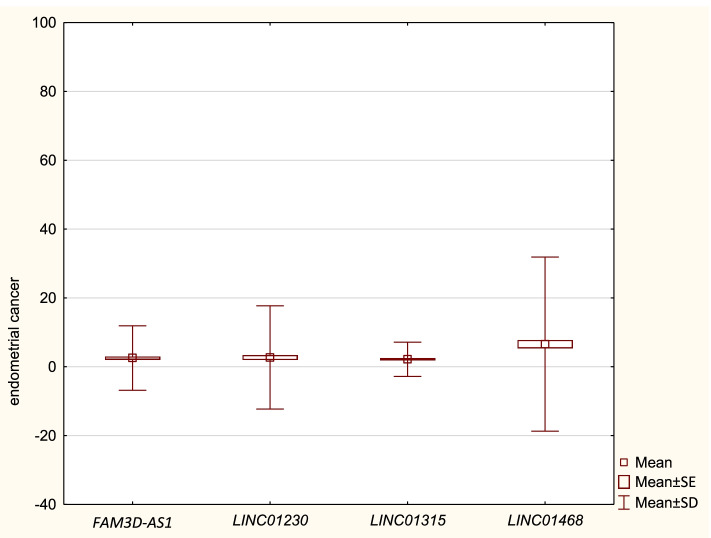
Expression of lncRNA sequences in Cases. Bars represent the mean values of Relative Quantification of studied lncRNA sequences’ expression in endometrial cancer tissue

**Fig. 2:**
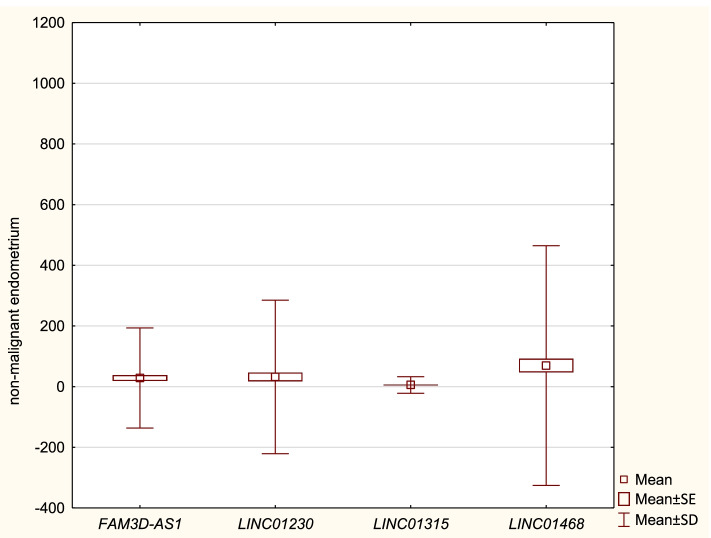
Expression of lncRNA sequences in Controls. Bars represent the mean values of Relative Quantification of studied lncRNA sequences’ expression in non-malignant endometrium

**Fig. 3:**
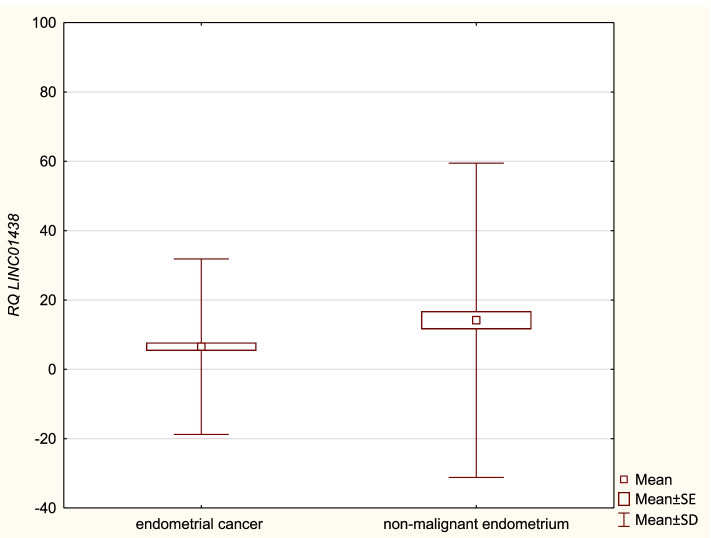
Expression of *LINC0148* in Cases and Controls, Relative Quantification (RQ). Bars represent the mean values of Relative Quantification of the expression of *LINC0148* in endometrial cancer and in non-malignant endometrial tissue. A statistically significant lower expression of *LINC0148* was revealed in endometrial cancer patients (Cases)

Clinical and pathological data of Cases and Controls are presented in Table 1. The expression of lncRNA *FAM3D-AS1*, *LINC01230*, *LINC01315* and *LINC01468* in Cases and Controls, measured by Relative Quantification (RQ), are presented in Tables (2 and 3) and Figs. (1 and 2), respectively. Our study revealed no statistically significant differences in expression of lncRNA sequences: *FAM3D-AS1*, *LINC01230* and *LINC01315* between EC patients and Controls. However, we have revealed a statistically significant lower expression of *LINC0148* in Cases (RQ: 6,56) in comparison to Controls (RQ: 69,46) - see Fig. 3. Table 4 presents the comparisons of expressions of the studied lncRNA in Cases and Controls.

**Table 1:** Clinical and pathological data of Cases and Controls

	Cases	Controls
Median age (range)	61,2 (47–85)	53,2 (48–84)
Body mass index (BMI) (kg/m^2^)		
< 24,9 (normal)	27 (22,5%)	29 (36,25%)
25–29,9 (overweight)	31 (25,8%)	28 (35%)
> 30 (obese)	62 (51,67%)	23 (28,75%)
Parity		
1	41 (34,17%)	22 (27,5%)
2 to 3	48 (40%)	44 (55%)
> 4	31 (25,83%)	14 (17,5%)
Use of hormone replacement therapy (HRT)		
Yes	23 (19,17%)	14 (17,5%)
No	97 (80,83%)	66 (82,5%)
Grading		
G1	44 (36,67%)	n/a
G2	58 (48,33%)	
G3	18 (15%)	
Staging (FIGO)		n/a
IA	71 (59,17%)	
IB	29 (24,17%)	
II	1 (0,83%)	
IIIA	5 (4,17%)	
IIIB	4 (3,33%)	
IIIC1	10 (8,33%)

**Table 2:** Expression of lncRNA sequences in Cases

lncRNA	N	RQ mean	RQ min.	RQ max.	SD
*FAM3D-AS1*	120	2,53	0,055	34,35	4,68
*LINC01230*	120	2,69	0,001	69,82	7,50
*LINC01315*	120	2,18	0,083	14,79	2,48
*LINC01468*	120	6,56	0,074	82,34	12,65

**Table 3:** Expression of lncRNA sequences in Controls

lncRNA	N	RQ mean	RQ min.	RQ max.	SD
*FAM3D-AS1*	80	28,60	0,044	380,21	82,45
*LINC01230*	80	32,24	0,044	949,85	126,47
*LINC01315*	80	5,67	0,007	61,85	13,64
*LINC01468*	80	69,46	0,105	911,30	197,55

**Table 4:** Correlation of expressions of lncRNA of the studied lncRNA sequences in Cases and Controls, Mann–Whitney *U* test, *p < 0,05*

lncRNA	p	N - Cases	N - Controls
*FAM3D-AS1*	0,135	120	80
*LINC01230*	0,074	120	80
*LINC01315*	0,080	120	80
*LINC01468*	**0,031**	120	80

**Fig. 4:**
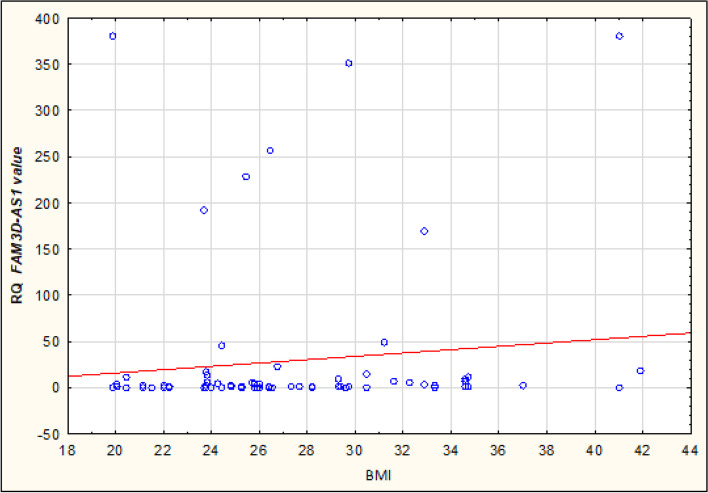
Correlation between *FAM3D-AS1* and Body Mass Index (BMI) in Controls. The graph represents the distribution of Relative Quantification of *FAM3D-AS1* in cancer-free patients. In our study *FAM3D-AS1* was positively correlated with Controls’ BMI

**Fig. 5:**
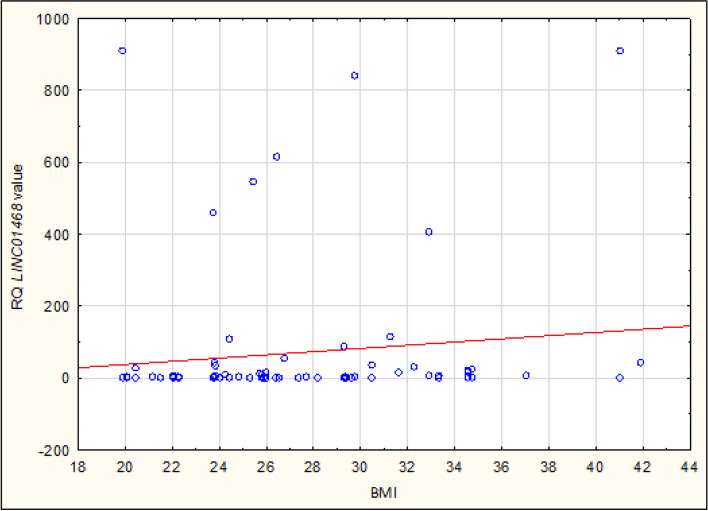
Correlation between *LINC01468* and Body Mass Index (BMI) in Controls. The graph represents the distribution of Relative Quantification of *LINC01468* in cancer-free patients. In our study *LINC01468* was positively correlated with Controls’ BMI

Expression of lncRNA sequences: *FAM3D-AS1*, *LINC01230*, *LINC01315* and *LINC01468* has been also analyzed in terms of correlation with clinical and pathological data of Cases: age, Body Mass Index (BMI), parity, use of hormone replacement therapy (HRT), cancer Grading and Staging - no statistically significant correlation was observed here. Interestingly, the study revealed a statistically significant correlation between the Body Mass Index (BMI) and two lncRNA sequences within Controls: both *FAM3D-AS1* and *LINC0148* are positively correlated with Controls’ BMI: R – Spearman: 0,252 and 0,260, respectively (see Table 5). Table 5 and Figs. (4 and 5) present the correlation of *FAM3D-AS1* and *LINC0148* with BMI. The other two studied sequences – *LINC01230* and *LINC01315* – presented no meaningful correlation with BMI in Controls. Alike, no correlation was observed between clinical and pathological data (age, BMI, parity, use of hormone replacement therapy) and studied lncRNA sequences in Controls.

**Table 5:** Correlation between studied lncRNA sequences and Body Mass Index (BMI) in Controls, Spearman’s rank correlation coefficient, *p < 0,05*

Variables	N -Controls	R- Spearman	*p*
*FAM3D-AS1* & BMI	**80**	**0,252**	**0,024**
*LINC01230* & BMI	80	0,202	0,073
*LINC01315* & BMI	80	0,109	0,335
*LINC01468* & BMI	**80**	**0,260**	**0,020**

## Discussion

In the Introduction we have presented a wide assortment of current literature data to deliver compelling evidence that transcription of lncRNA plays a considerable role in key biological processes in cancer. Some sequences of this non-coding transcripts have been already studied in endometrial cancer, but this area of research still stands mostly unexplored.

In our study we have presented an analysis of expression of *FAM3D-AS1*, *LINC01230*, *LINC01315* and *LINC01468* in EC patients compared to non-cancerous Controls with an eventual aim to correlate these lncRNA with the risk of this malignancy as well as with clinical and pathological data of studied Cases and Controls. According to the available body of literature, these sequences have not been yet investigated in endometrial cancer. Hence, our research is an original and unprecedented attempt to provide new data on the significance of these transcripts in EC.

Three out of four studied by us sequences (*FAM3D-AS1*, *LINC01230*, *LINC01315*) were found to be statistically irrelevant in terms of endometrial cancer risk and all clinical and pathological data presented in Table 1. It is worthwhile to mention, that the literature data on *FAM3D-AS1* and cancer is scarce, and all that is known now is that it may inhibit the development of colorectal cancer and that it is related to the cancer of pharynx and larynx [36, 37]. Yet, potential issues have been raised over the scientific validity of the former finding [48]. In a wider context, one must bear in mind, that colorectal and endometrial cancers - in some cases - share a common background: Lynch Syndrome [49]. The available data on other two sequences (*LINC01230* and *LINC01315*) is not abundant either. Although as little as 5 original papers on the latter are to be found in PubMed database and they cover a spectrum of some cancers there is no research data whatsoever on the role of the former sequence in cancer.

The only lncRNA sequence where our statistical analysis revealed a meaningful correlation with endometrial cancer was *LINC01468*. In Cases this sequence showed a clear downregulation which may suggest that it acts as a protective factor against this malignancy. Yet, *LINC01468* lacked statistical significance in terms of interrelationship with clinical and pathological data of Cases. Although this sequence has not been studied in endometrial cancer, there are other long non-coding transcripts that are not only related to EC itself, but to patients’ clinical and pathological features, i.e. Staging [50]. It only encourages to draw a conclusion that the investigation of selected lncRNA sequences is supported by convincing rationale and research in this field should continue.

Upon extended statistical analysis of our data, we have revealed a positive correlation between two lncRNA sequences (*FAM3D-AS1* and *LINC01468*) and Body Mass Index in Controls. This is an interesting finding if one considers that our non-cancerous control group consisted of patients operated on in our Institution for symptomatic uterine fibroids. Despite the lack of proper literature data on the abovementioned non-coding sequences in uterine fibroids alone, there is some evidence that confirms the positive correlation between obesity and uterine fibroids [51, 52]. But how in detail and why *FAM3D-AS1* and *LINC01468* are in our study statistically correlated within BMI-adjusted subgroups of our Controls – remains unclear.

However, there are some limitations of our study that need to be mentioned and considered with criticism. The dominant shortcoming of our analysis are numbers: a genetic research study on 120 Cases and 80 Controls (800 assays in total) makes an experienced researcher draw final conclusions with care and skepticism. Especially in genetics, our groups may be simply quantitatively unsatisfactory to conduct a fair judgement. Secondly, the Cases and Controls are not exactly homogenous – to some extent they differ by age, parity, BMI and use of hormone replacement therapy which may bias the results. Moreover, Cases do not constitute a group of disease-free women, but they were all treated surgically due to a benign gynecological condition – symptomatic uterine fibroids. Some reports suggest that some lncRNA sequences (i.e. lncRNA *SRA1* and *H19*) may exhibit a correlation with uterine fibroids’ tissue as such [53, 54], yet in our study the genetic assays were performed strictly on the selected endometrial tissue samples and not on fibroids nor myometrial tissue themselves.

Thirdly, still very little is known on the role of this non-coding ‘transcriptional noise’ and how to thoughtfully design a study to draw final and defensible conclusions. To bring limitations to a close, one needs to point out, that this analysis involved only endometrioid endometrial cancer with type II (non-endometrioid cancer) listed as the main exclusion criterion. Though, it is the latter that remains a real clinical challenge for healthcare providers in gynecological oncology.

Having in mind all the abovementioned findings and aware of the restrictions of our study, we dare to claim that this research has shed some new light on lncRNA in endometrial cancer and contributes to the growing – but still fragmentary – body of knowledge on these non-coding sequences in gynecological oncology.

## Conclusions

*LINC01468* may be a protective factor in endometrial cancer. The current state of knowledge on lncRNA in cancer, especially in endometrial cancer, is still limited. Further studies are warranted to further explore this subject.

## Supplementary Information

**Additional file 1.**

## Data Availability

All data generated or analyzed during this study are included in its supplementary information files.

## Abbreviations

lncRNA: Long non-coding RNA
BMI: Body Mass Index
EC: Endometrial cancer
IARC: International Agency for Research on Cancer
*SNHG12*: Small nucleolar RNA host gene 12
*H19*: Human H19 gene
*OVAAL*: Ovarian adenocarcinoma amplified lncRNA
*CASC2*: Cancer susceptibility candidate 2
*MALAT1*: Metastasis-associated lung adenocarcinoma transcript 1
*HOTAIR*: HOX transcript antisense RNA
*SRA*: Steroid receptor RNA activator
*Linc-RoR*: Long intergenic non-coding ribonucleic acids-regulator of programming
FFPE tissue sample: Formalin-Fixed Paraffin-Embedded tissue sample
